# Distinctive Structural and Molecular Features of Myelinated Inhibitory Axons in Human Neocortex

**DOI:** 10.1523/ENEURO.0297-18.2018

**Published:** 2018-10-16

**Authors:** Kristina D. Micheva, Edward F. Chang, Alissa L. Nana, William W. Seeley, Jonathan T. Ting, Charles Cobbs, Ed Lein, Stephen J Smith, Richard J. Weinberg, Daniel V. Madison

**Affiliations:** 1 Department of Molecular and Cellular Physiology, Stanford University, Stanford, CA, 94305; 2 Department of Neurological Surgery, University of California San Francisco, San Francisco, CA, 94143; 3 Memory and Aging Center, Department of Neurology, University of California San Francisco, San Francisco, CA, 94143; 4 Department of Pathology, University of California San Francisco, San Francisco, CA, 94143; 5 Cell Types Program, Allen Institute for Brain Science, Seattle, WA, 98109; 6 The Ben and Catherine Ivy Center for Advanced Brain Tumor Treatment, Swedish Neuroscience Institute, Seattle, WA, 98122; 7 Department of Cell Biology and Physiology, University of North Carolina, Chapel Hill, NC 27599

**Keywords:** array tomography, GABA, myelin

## Abstract

Numerous types of inhibitory neurons sculpt the performance of human neocortical circuits, with each type exhibiting a constellation of subcellular phenotypic features in support of its specialized functions. Axonal myelination has been absent among the characteristics used to distinguish inhibitory neuron types; in fact, very little is known about myelinated inhibitory axons in human neocortex. Here, using array tomography to analyze samples of neurosurgically excised human neocortex, we show that inhibitory myelinated axons originate predominantly from parvalbumin-containing interneurons. Compared to myelinated excitatory axons, they have higher neurofilament and lower microtubule content, shorter nodes of Ranvier, and more myelin basic protein (MBP) in their myelin sheath. Furthermore, these inhibitory axons have more mitochondria, likely to sustain the high energy demands of parvalbumin interneurons, as well as more 2’,3’-cyclic nucleotide 3’-phosphodiesterase (CNP), a protein enriched in the myelin cytoplasmic channels that are thought to facilitate the delivery of nutrients from ensheathing oligodendrocytes. Our results demonstrate that myelinated axons of parvalbumin inhibitory interneurons exhibit distinctive features that may support the specialized functions of this neuron type in human neocortical circuits.

## Visual Abstract

**Figure:**
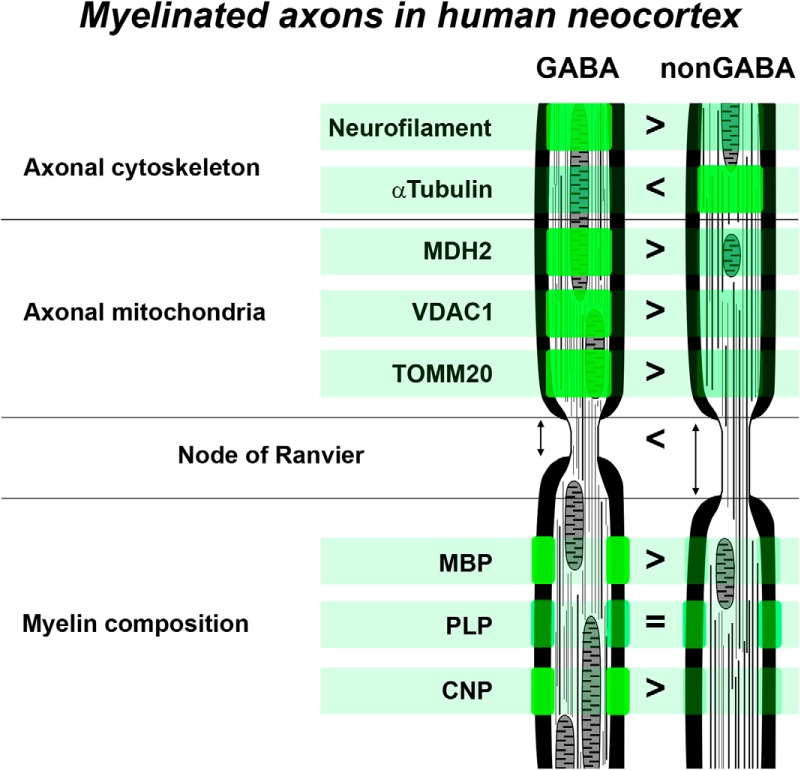

## Significance Statement

Numerous myelinated axons traverse the human neocortex, enabling fast and efficient signal transmission. Myelinated axons originate from both excitatory and inhibitory neurons, but their properties have rarely been studied in relation to parent neuron type. Here, we show that axons of inhibitory neurons have distinctive structural and molecular features that contrast with those of the majority of excitatory myelinated axons in human neocortex. These differences are likely to have important implications for neurologic disorders that involve pathologies of myelinated axons. For example, the distinct molecular and structural organization of inhibitory and excitatory myelinated axons may underlie differences in their vulnerability in neurologic disorders or to injuries, and may require different strategies for prevention and treatment.

## Introduction

Myelinated axons, which account for approximately half the volume of the human brain (Filley and Fields, 2016; Wandell, 2016), enable the dense, rapid, and efficient signal transmission central to human cognitive capacities. The insulating myelin sheath, created by a complex interaction between neuronal and glial cells, keeps axonal impulse propagation velocity high and energy consumption low, while allowing very small overall fiber diameters.

Myelinated axons convey information from a variety of neuronal types, and are likely to exhibit specialized features; yet, their properties have rarely been studied in relation to cell type (Gärtner et al., 2001; Jinno et al., 2007; Tomassy et al., 2014; Micheva et al., 2016; Stedehouder et al., 2017). Thus, very little is known about the diversity of CNS myelinated axons. Most of the available evidence comes from studies in rodents. Recently, it was shown that a large fraction of myelin in the mouse neocortex ensheathes axons of inhibitory neurons, specifically of parvalbumin-positive basket cells (Micheva et al., 2016). These inhibitory myelinated axons differ significantly from the excitatory axons in structural organization (e.g., length of nodes of Ranvier and internodes), and in molecular organization (e.g., cytoskeletal composition and protein content of myelin). Parvalbumin-positive myelinated axons have also been observed in human neocortex (Chung et al., 2013), and a recent study showed that the majority of cortical fast-spiking basket cells form myelinated axons (Stedehouder et al., 2017).

Do inhibitory myelinated axons in human cortex share the distinctive features reported in mouse cortex? There are no available data regarding their relative abundance, laminar distribution, or possible differences from excitatory myelinated axons. In the present study, we address these questions, using array tomography on surgically excised human neocortical tissue.

## Materials and Methods

### Human surgical specimens

Human neocortical tissue specimens were obtained during neurosurgeries for epilepsy treatment or tumor removal (Table 1). It was necessary to remove the overlying neocortical tissue to gain access to the diseased tissue. Before surgery, informed consent was obtained for the use of neurosurgical tissue for research purposes under protocols approved by the institutional review board of the University of California, San Francisco, and Swedish Medical Center, Seattle.

**Table 1.** Human samples

Patient	Gender	Age	Clinical diagnosis	Region	Fixative
Q1010	Male	30	Epilepsy	Anterior lateral temporal cortex	2% PFA, 2% GA in PB
Q1011	Male	44	Epilepsy	Anterior medial temporal cortex	4% PFA, 1% GA, 2.5% DMSO in PB
Q1014	Female	48	Epilepsy	Anterior lateral temporal cortex	2% PFA, 4% GA, 2.5% DMSO in CB
Q1015	Female	66	Epilepsy	Anterior lateral temporal cortex	2% PFA, 4% GA, 2.5% DMSO in CB
Q1016	Male	49	Epilepsy	Lateral temporal cortex	2% PFA, 4% GA, 2.5% DMSO in CB
Q1017	Male	46	Epilepsy	Lateral temporal cortex	2% PFA, 4% GA, 2.5% DMSO in CB
Q1018	Female	20	Epilepsy	Lateral temporal cortex	2% PFA, 4% GA, 2.5% DMSO in CB
Q1019	Female	51	Epilepsy	Lateral temporal cortex	2% PFA, 4% GA, 2.5% DMSO in CB
10/16/14	Female	30	Nonspecific inflammation of the white matter	Frontal cortex	4% PFA in PB

CB, cacodylate buffer; DMSO, dimethyl sulfoxide; GA, glutaraldehyde; PB, phosphate buffer; PFA, paraformaldehyde.

Resected cortical samples from neurosurgeries for epilepsy treatment were rinsed in saline and placed in room temperature fixative for 1 h. The tissue was further fixed for 23 h at 4°C, for a total time of 24 h in fixative. The average time between resection and placement in fixative was 8 min, with a range of 4–18 min. The tissue was then transferred to PBS with 0.01% sodium azide and stored at 4°C before further processing.

### Array tomography

The tissue was dehydrated and embedded using standard array tomography protocols (Micheva et al., 2010). Small tissue chunks from neocortex were dissected out and rinsed in PBS with 50 mM glycine at 4°C (three changes for up to 30 min total). The tissue was then dehydrated in graded series of ethanol (50%, 70%, 95%, 100%, 100%), followed by a mixture of equal amounts of LRWhite and 100% ethanol, and finally three changes in 100% LRWhite. Each step was done for 10 min at 4°C. After that, the samples were left for 24 h at 4°C in LRWhite for complete infiltration, then transferred to gelatin capsules filled with LRWhite, and polymerized for 24 h in an oven set at 55°C. The polymerized blocks with tissue were stored at room temperature. Additionally, separate tissue chunks from three of the samples (Q1010, Q1011, Q1014) were prepared by freeze-substitution and embedding in Lowicryl HM20, following the protocol reported in Micheva et al. (2016). This tissue was used for the experiments with mitochondrial markers.

To prepare ribbons of serial sections, the blocks were trimmed around the tissue to the shape of a trapezoid, and glue (Weldwood Contact Cement diluted with xylene) was applied with a thin paint brush to the leading and trailing edges of the block pyramid. The embedded plastic block was cut on an ultramicrotome (Leica Ultracut EM UC6) into 70-nm-thick serial sections, which were mounted on gelatin-coated coverslips.

### Mouse tissue

Six adult mice (three to seven months old) were used. All animal procedures were performed according to National Institutes of Health and University of North Carolina guidelines. Three of these mice were used for a previous study (Micheva et al., 2016). The mice were perfusion-fixed with 2% glutaraldehyde and 2% formaldehyde in phosphate buffer, freeze substituted, and embedded in Lowicryl HM20, as described in Micheva et al. (2016). Array tomography was performed as described above for the human tissue. Somatosensory cortex (the three mice from Micheva et al., 2016) and visual cortex (the additional three mice) were analyzed.

### Immunofluorescence

Sections were processed for standard indirect immunofluorescence, as described in Micheva et al. (2010). Antibodies were obtained from commercial sources and are listed in Table 2. Array tomography-specific controls are presented in Extended Data Table 2-1, Figure 2-2. The antibodies against GABA, parvalbumin, α-tubulin, and neurofilament heavy chain have previously been characterized for array tomography (Bennett and Brody, 2015; Micheva et al., 2016) and gave the expected characteristic tissue staining patterns; therefore, they are not included in the analysis in Extended Data Table 2-1. The sections were pretreated with sodium borohydride [1% in Tris-buffered saline (TBS), pH 7.6 for 3 min] to reduce non-specific staining and autofluorescence. After a 20-min wash with TBS, the sections were incubated in 50 mM glycine in TBS for 5 min, followed by blocking solution (0.05% Tween 20 and 0.1% BSA in TBS) for 5 min. The primary antibodies were diluted in blocking solution as specified in Table 2 and were applied for 2 h at room temperature or overnight at 4°C. After a 15-min wash in TBS, the sections were incubated with Alexa Fluor dye-conjugated secondary antibodies, highly cross-adsorbed (Life Technologies), diluted 1:150 in blocking solution for 30 min at room temperature. Finally, sections were washed with TBS for 15 min, rinsed with distilled water, and mounted on glass slides using SlowFade Gold Antifade Mountant with DAPI (Invitrogen). After the sections were imaged, the antibodies were eluted using a solution of 0.2 M NaOH and 0.02% SDS, and new antibodies were reapplied. Several rounds of elution and restaining were applied to create a high-dimensional immunofluorescent image.

**Table 2:** Antibodies used in the study

Antigen	Host	Antibody source	Dilution	RRID
MBP	Chicken	AVES MBP	1:200	RRID:AB_2313550
GABA	Guinea pig	Millipore AB175	1:5000	RRID:AB_91011
Parvalbumin	Rabbit	SWANT PV28	1:300	RRID:AB_2315235
Neurofilament heavy	Chicken	AVES NFH	1:100	RRID:AB_2313552
α-Tubulin	Rabbit	Abcam ab18251	1:100	RRID:AB_2210057
PLP	Chicken	AVES PLP	1:100	RRID:AB_2313560
CNPase	Chicken	AVES CNP	1:100	RRID:AB_2313538
MDH2	Rabbit	Origene TA308153	1:200	RRID:AB_2722674
TOMM20	Rabbit	Abcam ab78547	1:100	RRID:AB_2043078
VDAC1	Rabbit	ProteinTech 10866-1-AP	1:50	RRID:AB_2257153

Antibody controls are presented in Extended Data Table 2-1, Figure 2-2. RRID, Research Resource Identifier; MBP, myelin basic protein; PLP, proteolipid protein; CNPase, 2’,3’-cyclic nucleotide 3’-phosphodiesterase; MDH2, malate dehydrogenase 2; TOMM20, translocase of outer mitochondrial membrane 20; VDAC1, voltage dependent anion channel 1.

10.1523/ENEURO.0297-18.2018.t2-1 Extended Data Table 2-1 **Antibody controls: Pearson’s correlation (PC) coefficients from four different control experiments.** The comparison between adjacent sections tests the consistency of staining, as the distribution of targets is very similar on two adjacent ultrathin sections (70-nm thickness). This correlation is influenced by antibody characteristics, but also the size of targets, with smaller targets displaying larger spatial variability from section to section, and therefore a smaller PC coefficient. The comparison with an antibody against an overlapping antigen is a test for the specificity of staining. The following comparisons were done: MBP/PLP, MBP/CNP, MDH2/TOMM20, MDH2/VDAC1. The lower PC coefficient for CNP reflects the fact that CNP is present only in certain regions of the myelin sheath, while MBP is an integral protein of myelin. The antibodies against mitochondrial proteins (MDH2, TOMM20, and VDAC1) also have lower PC coefficients, because TOMM20 and especially VDAC1 antibodies give a rather sparse punctate immunolabeling of mitochondria (Fig. 4; Extended Data Figure 2-2), and even when labeling the same mitochondrion, the immunofluorescence signal from the different antibodies often does not overlap. Another test for specificity is the comparison with an antibody against a spatially exclusive antigen. PC coefficient values of 0 and below are expected in this case. MBP, PLP, and CNP (present in the myelin sheath) were each compared with GABA (inside inhibitory neurons); MDH2, TOMM20, and VDAC1 (mitochondria) were compared with MBP (myelin sheath). And finally, all antibodies were compared with DAPI to control for background nuclear staining. Download Table 2-1, DOCX file.

10.1523/ENEURO.0297-18.2018.f2-2 Extended Data figure 2-2 **Immunolabeling of mitochondria on LRWhite sections from mouse neocortex.** Each image is a MAX projection from four serial sections, 70 nm each. The grey image is the tissue autofluorescence imaged in the 488 channel, and it is superimposed with the immunofluorescence from a mitochondrial marker in green (left column) or with the DAPI label of nuclei in blue (right column). Arrowheads point to several mitochondria, which can be seen as brighter elongated structures within the neuronal cell bodies. There are also abundant mitochondria within the neuropil, but they are harder to distinguish in the autofluorescence image. Download Figure 2-2, TIF file.

The immunostained ribbons of sections were imaged on an automated epifluorescence microscope (Zeiss AxioImager Z1) using a 63× Plan-Apochromat 1.4 NA oil objective. To define the position list for the automated imaging, a custom Python-based graphical user interface, MosaicPlanner (obtained from https://code.google.com/archive/p/smithlabsoftware/), was used to automatically find corresponding locations across the serial sections. Images from different imaging sessions were registered using a DAPI stain present in the mounting medium. The images from the serial sections were also aligned using the DAPI signal. Both image registration and alignment were performed with the MultiStackReg plugin in FIJI (Schindelin et al., 2012).

### Immunofluorescent image analysis and statistics

For each sample, we used volumes spanning at least four cortical layers. In five of the samples, all layers were included. For each layer, a field of view of ∼135 × 100 μm was imaged and analyzed. The volumes comprised of 33 serial sections on average (range of 12–67 sections). Immunofluorescence measurements were performed on raw images using FIJI. Myelin basic protein (MBP) or proteolipid protein (PLP) immunofluorescence were used to define regions of interest (ROIs), which were either the myelin sheath for measurements of MBP, PLP, and 2’,3’-cyclic nucleotide 3’-phosphodiesterase (CNP) signals, or the axon under the myelin sheath for measurement of axonal immunofluorescence for GABA, parvalbumin (PV), cytoskeletal and mitochondrial proteins. The mean gray value of immunolabels was compared between GABA and nonGABA, or PV and nonPV axons from the same coverslip, using the nonparametric Mann–Whitney *U* test, with statistical significance set to *p* ≤ 0.01. To prepare the boxplots, the web application BoxPlotR was used (http://boxplot.tyerslab.com; Spitzer et al., 2014).

## Results

The presence and distribution of myelinated axons was quantified in samples from human temporal neocortex, using array tomography. The tissue samples were obtained from surgical resections for epilepsy, immersion fixed with a mixture of paraformaldehyde and glutaraldehyde, and embedded in resin (Table 1). Serial ultrathin (70 nm) sections were cut from the tissue blocks, immunostained for the inhibitory neurotransmitter GABA, MBP, and other relevant markers (Table 2), and imaged with a fluorescence microscope.

**Figure 1.**
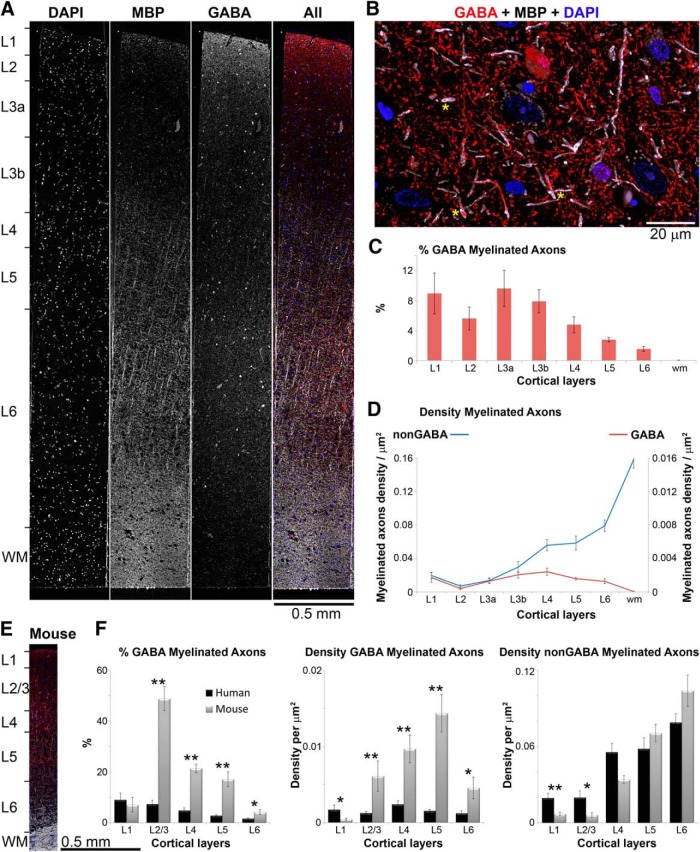
Distribution of inhibitory GABA myelinated axons in human temporal cortex. ***A***, A 70-nm-thick section through human cortex immunostained with MBP (white) and GABA (red). Nuclei are labeled with DAPI (blue). ***B***, Volume reconstruction of a subregion from layer 3a (35 serial sections, 70 nm each). Several GABA myelinated axons are marked with yellow asterisks. ***C***, Proportion of myelinated axons that contain GABA in human temporal cortex. Means and SEs from eight human patients are shown. ***D***, Density of nonGABA and GABA myelinated axonal profiles. Means and SEs from eight human patients are shown. ***E***, A 70-nm-thick section through mouse cortex (MBP, white; GABA, red; and DAPI, blue) shown at the same scale as the human cortical section in panel ***A***. Note the difference in cortical thickness between the two species. ***F***, Comparison of the proportion of GABA myelinated axons and the density of myelinated axons throughout the layers of human and mouse cortex (means and SEs from eight human patients and six mice are shown). For this comparison, human results for layers 2, 3a, and 3b were pooled together to compare with mouse cortical layer 2/3. *p < 0.05, **p ≤ 0.01.

MBP immunofluorescence clearly outlines myelinated axons, some of which contain the inhibitory neurotransmitter GABA (Fig. 1). Myelinated GABA axonal profiles are observed in all human cortical layers at a density of 0.001–0.002 per μm^2^, with the exception of layer 2, where they are significantly sparser (*p* < 0.01 compared to layers 1, 3b, 4, and 5, and *p* < 0.05 compared to layers 3a and 6). Myelinated GABA axons are rarely encountered in subcortical white matter. The proportion of myelinated axons that contain GABA decreases in cortical layers 4, 5, and 6, as the density of nonGABA myelinated axons substantially increases with cortical depth. Overall, the proportion of myelinated axons that contain GABA is significantly lower in the human temporal cortex compared to mouse somatosensory and visual cortex (Fig. 1*E*,*F*). For example, the highest percentage of GABA myelinated axons in human cortex is in layer 3a, 10 ± 2%, whereas GABA myelinated axons constitute 48 ± 3% of axons in mouse cortical layer 2/3 (Micheva et al., 2016). This is mostly due to the lower density of GABA myelinated axons in human cortex, while the density of nonGABA myelinated axons in not significantly different between human and mouse cortex in all layers, except layer 1 (*p* < 0.01). Layer 1 is also the only cortical layer, where the density of GABA myelinated axons is higher in human cortex compared to mouse (*p* = 0.012).

**Figure 2.**
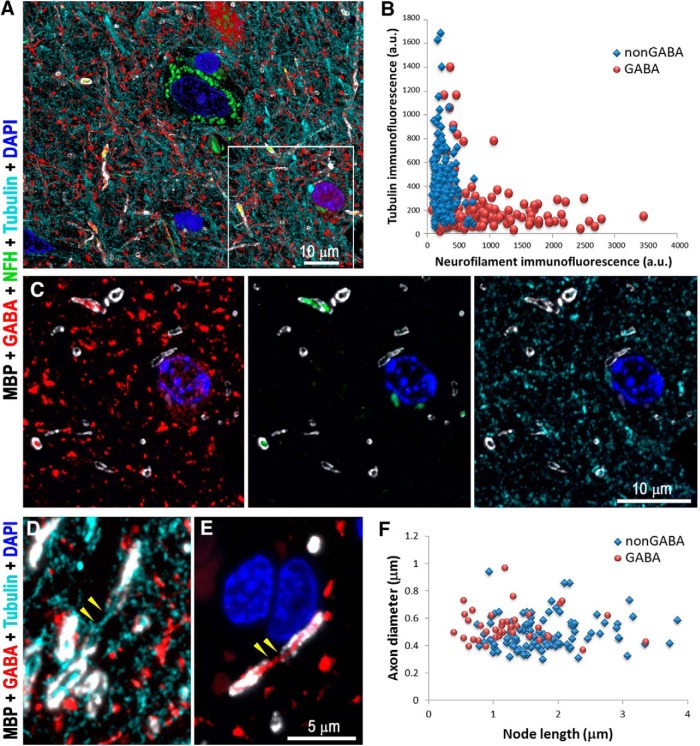
Myelinated GABA axons have distinct cytoskeletal composition and shorter nodes of Ranvier, compared to nonGABA myelinated axons. ***A***, Volume reconstruction of 35 serial sections (70 nm each) from layer 3a of human cortex immunolabeled with GABA (red), MBP (white), neurofilament heavy chain (green), and α-tubulin (cyan). A single section from the boxed region is shown in ***C***. ***B***, Analysis of the cytoskeletal content of myelinated GABA versus nonGABA axons from layers 3a, 3b, 4, and 5 from three human samples (216 nonGABA and 164 GABA myelinated axons). ***C***, A single section from the boxed region in ***A***, showing different combinations of immunostains. Note that myelinated GABA axons are brightly labeled with the neurofilament antibody but have weak tubulin immunoreactivity. ***D***, Maximum projection from three serial sections showing a node of Ranvier (yellow arrowheads) from a nonGABA axon. At the node, which is devoid of MBP immunofluorescence, the axon can be followed using the tubulin immunofluorescence (cyan). ***E***, Node of Ranvier (yellow arrowheads) from a GABA axon. ***F***, Comparison of the lengths of the nodes of Ranvier from GABA and nonGABA cortical axons (39 GABA and 100 nonGABA nodes, eight samples).

A previous study of mouse cortex reported a number of differences between myelinated inhibitory and excitatory axons, which were also seen in human cortex (Fig. 2). Myelinated GABA axons in human cortex have significantly higher neurofilament content (average immunofluorescence of 810 ± 49 average units (a.u.) vs 248 ± 9 a.u. for nonGABA myelinated axons, *p* < 0.00001) and lower tubulin content compared to myelinated nonGABA axons (immunofluorescence of 222 ± 17 vs 413 ± 17 a.u., *p* < 0.00001; *n* = 164 GABA and *n* = 216 nonGABA myelinated axons from layers 3a through 5 from three different samples; Fig. 2*A–D*). The nodes of Ranvier (Rasband and Peles, 2015), which appear as short gaps in the MBP and PLP staining along myelinated axons, are significantly shorter for myelinated GABA axons compared to unlabeled, presumably excitatory, axons (1.28 ± 0.10 vs 1.79 ± 0.07 μm, *p* < 0.0001; *n* = 39 GABA and *n* = 100 nonGABA nodes from eight different samples, all cortical layers; Fig. 2*F*). Finally, GABA axons have more MBP in their myelin than do neighboring nonGABA axons (immunofluorescence of 1026 ± 41 vs 884 ± 29 a.u., *p* = 0.007; *n* = 164 GABA and *n* = 216 nonGABA myelinated axons from layers 3a through 5 from three different samples), although their PLP content is essentially the same (1708 ± 62 vs 1643 ± 48 a.u., *p* = 0.68). Thus, the distinctive characteristics of myelinated inhibitory axons seen in mouse are also present in human cortex.

**Figure 3.**
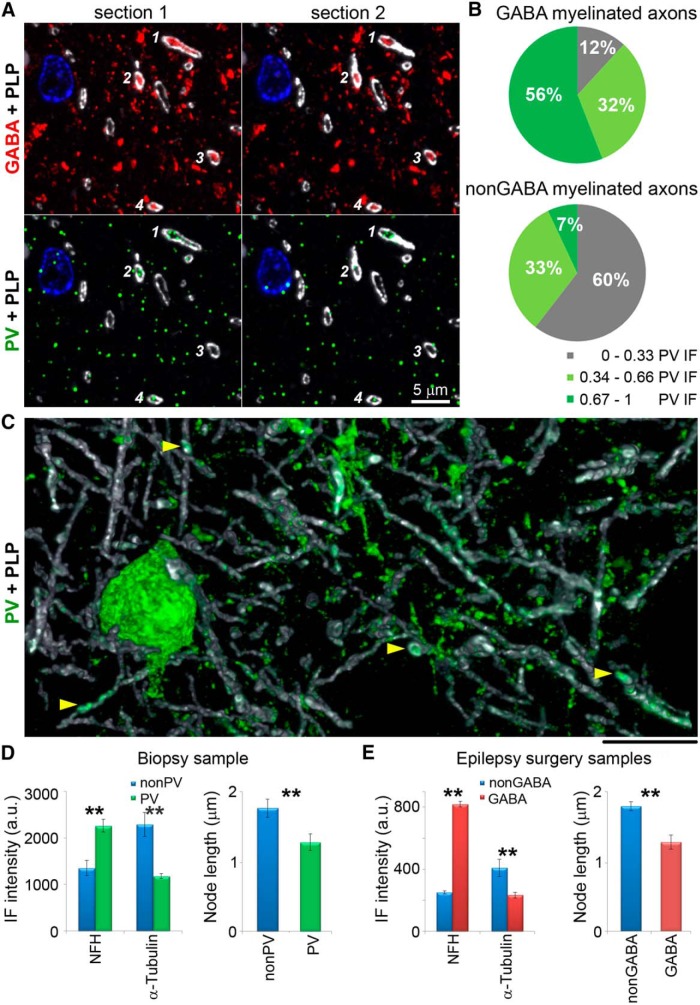
The majority of GABA myelinated axons are parvalbumin-immunopositive. ***A***, Two consecutive sections from layer 3b of human cortex, immunolabeled for GABA (red), parvalbumin (green), and MBP (white). Cell nuclei are stained with DAPI (blue). Four myelinated GABA axonal profiles are marked with numbers; three of them (1, 2, and 4) are immunoreactive for parvalbumin. ***B***, Pie charts showing the distribution of GABA and nonGABA myelinated profiles according to their parvalbumin immunoreactivity. Parvalbumin immunofluorescence was normalized for each sample (186 GABA and 172 nonGABA axonal profiles from two different samples). ***C***, PV neuron and processes in layer 3b of human cortex from a biopsy; volume reconstruction from 49 serial sections. Yellow arrowheads point to PV-positive myelinated axons. ***D***, Cytoskeletal composition and node length of myelinated axons in biopsy tissue. Left, Comparison of the immunofluorescence intensity (mean ± SE) for neurofilament heavy chain and α–tubulin within myelin profiles of nonPV (blue) and PV axons (green) from the biopsy sample. The differences are statistically significant (*p* < 0.001 for NFH and *p* < 0.0001 for α-tubulin; 48 nonPV and 26 PV axons from layers 3a, 3b, and 4 from one sample). Right, Nodes of PV axons are shorter than nonPV axons in the biopsy sample (*p* < 0.01; 34 nonPV and 24 PV nodes). ***E***, Cytoskeletal composition and node length of myelinated axons in epilepsy surgery tissue. Left, Comparison of the immunofluorescence intensity for neurofilament heavy chain and α–tubulin within myelin profiles of nonGABA (blue) and GABA axons (red) from epilepsy surgery samples. The differences are statistically significant (*p* < 0.0001, 196 nonGABA and 177 GABA axons from layers 3a, 3b, and 4 from three different samples). Right, Nodes of GABA axons are shorter than nonGABA axons in epilepsy surgery samples (*p* < 0.0001; 100 nonGABA and 39 GABA nodes from eight different samples). **p <0.01.

In mouse, the overwhelming majority of myelinated GABA axons come from parvalbumin-containing basket cells (Micheva et al., 2016). To determine the source of myelinated GABA axons in human cortex, we immunostained the samples with an antibody against parvalbumin. More than half of the myelinated GABA axons are clearly immunoreactive for parvalbumin, with PV-immunofluorescent signal present on consecutive sections (Fig. 3). On average, the PV immunofluorescence within GABA axons is significantly higher than within nonGABA axons (68 ± 2 vs 42 ± 1 a.u., *p* < 0.0001, *n* = 186 GABA and *n* = 172 nonGABA myelinated axons from two different samples). However, PV immunoreactivity in these samples is generally weak and displays a low signal-to-noise ratio, making it difficult to determine the total number of PV-positive myelinated GABA axons. Low and variable levels of parvalbumin have been previously described in cortex of patients with epilepsy (Marco and DeFelipe, 1997).

To confirm our findings, we also used cortical tissue from a non-epileptic patient who had overlying cortical tissue removed during a biopsy to diagnose an inflammatory lesion. In this cortical specimen, we observe numerous myelinated axons immmunoreactive for parvalbumin (Fig. 3*C*). The proportion of the myelinated PV axons is very similar to the GABA axons in the tissue from epilepsy surgeries. For example, in layer 3a, 15% of the myelinated axonal profiles are PV-immunopositive, and in layer 3b, 14% are PV-immunopositive (693 myelinated axons from layer 3a and 2019 from layer 3b). This is within the range observed for GABA myelinated axons from epilepsy surgeries (3–21% in layer 3a, and 3–15% in layer 3b). The biopsy tissue, however, was fixed only with paraformaldehyde, which prevented simultaneous labeling with an anti-GABA antibody, since glutaraldehyde is required for satisfactory preservation of GABA (Somogyi et al., 1985; Schiffmann et al., 1988). However, other characteristics of the PV-immunopositive myelinated axons from the non-epilepsy biopsy tissue, such as their cytoskeletal content and length of nodes of Ranvier (Fig. 3*D*,*E*), are also very similar to the GABA axons in the epilepsy surgery tissue, suggesting that these axon populations are comparable. Thus, parvalbumin interneurons appear to be a major source of inhibitory myelinated axons in human neocortex.

**Figure 4.**
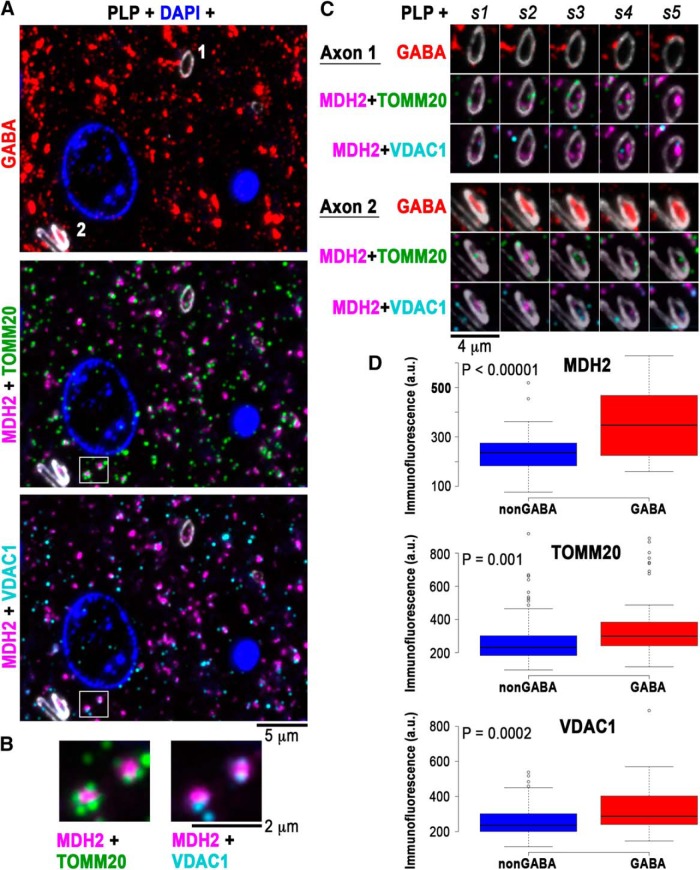
GABA myelinated axons have more mitochondria. ***A***, A single 70-nm section from layer 4 of human cortex immunolabeled with GABA (red), PLP (white), MDH2 (magenta), TOMM20 (green), and VDAC1 (cyan). ***B***, The boxed area in ***A*** is enlarged to show two immunolabeled mitochondria, with the MDH2 mitochondrial matrix protein surrounded by the outer mitochondrial membrane proteins TOMM20 and VDAC1. ***C***, Five serial sections through axons 1 and 2, marked on ***A***, top section. Axon 1 is nonGABA and axon 2 is GABA. Adjacent to axon 2, there is also a nonGABA axon. ***D***, Boxplots of the immunofluorescence for MDH2, TOMM20, and VDAC1 within nonGABA and GABA myelinated axons. Center lines show the medians, box limits indicate the 25th and 75th percentiles as determined by R software; whiskers extend 1.5 times the interquartile range from the 25th to the 75th percentiles, outliers are represented by dots, *n* = 88 and *n* = 41 axons for MDH2, *n* = 95 and *n* = 43 axons for TOMM 20, and *n* = 97 and *n* = 47 axons for VDAC1, from layers 3a through 5 from three different samples.

Parvalbumin-positive basket cells are fast-spiking interneurons capable of generating long trains of action potentials at very high frequency (Kawaguchi et al., 1987; Kawaguchi and Kubota, 1997), which requires a constant supply of ATP. Thus, the high energy demands of parvalbumin interneurons may be reflected in a higher content of mitochondria within their myelinated axons to ensure the local production of ATP. Their cell bodies (Gulyás et al., 2006; in rodent cortex), as well as presynaptic boutons (Cserép et al., 2018; in rodent and human cortex), are known to have abundant mitochondria, but the mitochondrial content of their axons has not been assessed. We used antibodies against three mitochondrial proteins: MDH2, which is present in the mitochondrial matrix, and TOMM20 and VDAC1, which are proteins of the outer mitochondrial membrane (Fig. 4). Accordingly, immunolabels for TOMM20 and VDAC1 were seen surrounding the MDH2 label (Fig. 4*B*). We quantified the distribution of these three mitochondrial markers in GABA and nonGABA myelinated axons in the human epilepsy surgery samples. The immunofluorescence for all three mitochondrial markers was significantly higher in myelinated GABA compared to nonGABA myelinated axons (MDH2, *p* < 0.00001; TOMM20, *p* = 0.001; VDAC1, *p* = 0.0002), indicating that myelinated GABA axons in human cortex are indeed enriched in mitochondria (Fig. 4).

**Figure 5.**
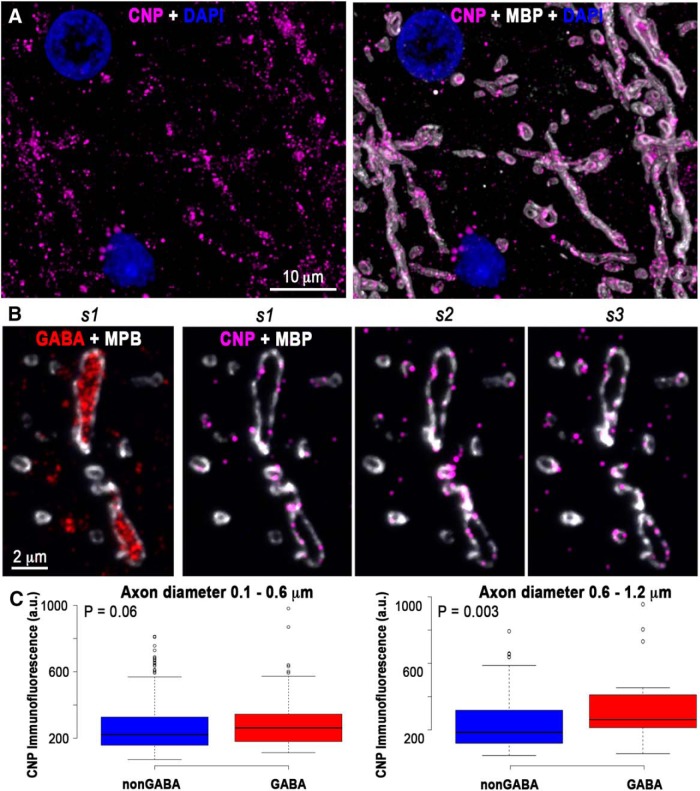
GABA myelinated axons have more CNP in their myelin. ***A***, Volume reconstruction of 21 serial sections from human cortical layer 5, immunostained with CNP (magenta) and MBP. ***B***, Three consecutive sections through a GABA myelinated axon immunolabeled for CNP (magenta) and MBP (white). The first section is also shown immunolabeled for GABA. ***C***, Thicker GABA axons (0.6–1.2 μm in diameter) have significantly more CNP in their myelin sheath compared to nonGABA axons of similar thickness. Center lines in boxplots show the medians, box limits indicate the 25th and 75th percentiles as determined by R software; whiskers extend 1.5 times the interquartile range from the 25th to the 75th percentiles, outliers are represented by dots. For the boxplot on the left (axon diameter 0.1–0.6 μm), *n* = 341 nonGABA and *n* = 98 GABA myelinated axons from two samples), and for the boxplot on the right (axon diameter 0.6–1.2 μm), *n* = 142 nonGABA and *n* = 34 GABA axons from two samples).

How do these axons receive the nutrients necessary for the mitochondrial production of ATP? Trophic support to myelinated axons in the CNS is likely provided through a system of cytoplasmic channels within myelin (for review, see Simons and Nave, 2015), whose maintenance requires the myelin protein CNP (Snaidero et al., 2017). Our experiments reveal that GABA myelinated axons have a significantly higher CNP content compared to nonGABA axons (321 ± 17 vs 271 ± 7 a.u.; *p* = 0.002, *n* = 133 GABA and *n* = 489 nonGABA myelinated axons) suggesting the existence of more cytoplasmic channels within their myelin, consistent with a higher need for trophic support. Interestingly, this difference in CNP content between GABA and nonGABA axons is most pronounced among myelinated axons with a diameter larger than 0.6 μm (Fig. 5).

## Discussion

Using samples of surgically excised brain tissue, we show here that many of the characteristic features of myelinated axons in cortical gray matter previously described in the mouse (Micheva et al., 2016) are also present in human, despite 100 million years of divergence. As in mouse, many human myelinated inhibitory axons originate from PV-positive interneurons and have distinctive features, including high neurofilament and low microtubule content, short nodes of Ranvier, and high content of MBP in their myelin sheath. The implications of these differences will require further study, but we speculate that they have direct functional significance. For example, the cytoskeleton is directly involved in axonal transport (Conde and Cáceres, 2009: Hirokawa et al., 2010); it also determines the mechanical properties of axons and therefore their susceptibility to mechanical injury (Ratzliff and Soltesz, 2000; Grevesse et al., 2015). The length of the nodes of Ranvier affects the speed and timing of action-potential propagation (Arancibia-Cárcamo et al., 2017). MBP is a target in multiple sclerosis (Allegretta et al., 1990) and differences in its content may influence the fate of axons in brain pathologies. We further show that the inhibitory myelinated axons have more mitochondria, as well as more CNP, a protein enriched in the myelin cytoplasmic channels that is thought to provide access for trophic support from ensheathing oligodendrocytes. This is consistent with the high energy demands of fast-spiking PV interneurons and suggests that, in addition to influencing conduction velocity, the myelination of inhibitory axons is likely beneficial for managing their energy consumption by increasing the efficiency of action potential propagation (Hartline and Colman, 2007) and providing trophic support (Fünfschilling et al., 2012; Lee et al., 2012).

There are also important differences between human and mouse neocortical myelinated axons. The density of GABA myelinated axons in human cortex is substantially less than in mouse cortex, while the density of nonGABA myelinated axons is rather similar, with the exception of layers 1 and 2. This may appear puzzling at first glance, because of the association of interneurons with high-level cortical functions (for review, see Tremblay et al., 2016). However, this is consistent with the lower densities of other neuronal structures in human cortex. For example, synapse density is ∼2.5–3 times less in human cortex compared to mouse, and neuron density is five times less (DeFelipe et al., 2002). This is likely due, at least in part, to the increased proportion and size of nonneuronal cells in human cortex (for review, see Herculano-Houzel, 2014). If the same scaling down of density applies to myelinated axons, then it would appear that GABA myelinated axons are following the general trend, while nonGABA myelinated axons have a disproportionately high density, possibly because of the much longer distances many of these axons have to cover in the human brain and therefore the advantages offered by myelination to speed up action potential propagation. One notable exception to the lower density of GABA myelinated axons in human cortex is layer 1, which has more GABA myelinated axons compared to mouse layer 1. Layer 1 is the site where input from a variety of cortical and subcortical neurons is integrated (Douglas and Martin, 2004; Harris and Shepherd, 2015) and which appears to have undergone large evolutionary changes. For example, recent studies revealed two new interneuron types in human cortex that are not present in mouse cortex, and both of these interneurons are enriched in layer 1 (Boldog et al., 2018; Hodge et al., 2018). The source of the GABA myelinated axons in human layer 1 remains to be determined and will provide further insight into the functional significance of this evolutionary change.

The characteristic features of myelinated inhibitory axons in human cortical gray matter are likely to have important implications for neurologic disorders that involve pathologies of myelinated axons. The distinct molecular and structural organization of inhibitory and excitatory myelinated axons may underlie differences in their vulnerability in neurologic disorders or to injuries. Furthermore, disturbances of myelin around inhibitory axons are expected to have very different functional implications compared to disturbances of myelin on excitatory axons and may require different strategies for prevention and treatment.

## Synthesis

Reviewing Editor: Quentin Pittman, University of Calgary Decisions are customarily a result of the Reviewing Editor and the peer reviewers coming together and discussing their recommendations until a consensus is reached. When revisions are invited, a fact-based synthesis statement explaining their decision and outlining what is needed to prepare a revision will be listed below. The following reviewer(s) agreed to reveal their identity: Dan Nicholson, Mikael Simons.

Myelin pattern has been investigated in the mouse cortex revealing interesting patchy distribution of myelin along the axon. In particular inhibitory neurons show a pattern of myelination that differs significantly from what we know about myelination in most brain regions. However, since myelination is the mouse cortex is slow and continuous for months, it is not clear whether these distinct patterns of myelin disappear with time. Using human adult samples and array tomography, the authors now show that many of the characteristic features of myelinated axons in cortical grey matter previously described in the mouse are also present in human. As in mouse, many human myelinated inhibitory axons originate from parvalbumin positive interneurons and have distinctive features, including high neurofilament, low microtubule content and high content of MBP.

Although this paper is largely a human version of a previous paper from the Smith/Bock labs, it is nonetheless an important small piece with direct human relevance. Also, the techniques are an advance to the field. Array tomography is incredibly useful, and is the only available technique with the powerful multiplexing capabilities. But, it gets scant attention compared to other super-resolution light microscopy techniques. So, this MS is a nice use of this incredibly powerful technique.

A) The major concern is with the statistics. The authors do not explain what comparisons were, how they were, whether they were significant, and what the n's were. They need to address this big concern. Also, are differences in the figures statistically significant? Why are there not asterisks indicating statistically reliable differences?

B) Only 10% of GABA positive axons are myelinated. The findings should not be overestimated and discussed accordingly

C) The discussion should address differences between mice and humans

D) Although not necessary for acceptance, if data were available, inclusion of the following would improve it:

1) The density of Mitochondria and Myelin could also be evaluated by EM

2) For the identification of nodes antibodies could be used.

3) Quantification of mitochondrial markers is done in layer 5, but the other analyses are performed in layer 3 and 4. The analysis could be extended.

## Author Response

Response to reviewers

Please see our responses to each comment below in blue. Thank you for helping us make this a better paper!

A) The major concern is with the statistics. The authors do not explain what comparisons were, how they were, whether they were significant, and what the n's were. They need to address this big concern. Also, are differences in the figures statistically significant? Why are there not asterisks indicating statistically reliable differences?

We have now included more details about statistics, including n's, p-values, and asterisks in figures to indicate significance. We also redid the statistical analysis using the nonparametric Mann-Whitney test to avoid complications due to possible deviations from normal distribution of the data. In Figures 4 and 5, we changed the bar plots to box-and-whisker plots, which are more informative.

B) Only 10% of GABA positive axons are myelinated. The findings should not be overestimated and discussed accordingly

This is indeed an important point and we have added a paragraph to the Discussion.

C) The discussion should address differences between mice and humans

We completely agree with the reviewers. We have now added panels to Figure 1 (E and F) to compare human and mouse data on myelinated axons and we have addressed the observed differences in a new paragraph in Discussion

D) Although not necessary for acceptance, if data were available, inclusion of the following would improve it:

1) The density of Mitochondria and Myelin could also be evaluated by EM

While we are not able to add EM data at this point, we performed additional IF experiments to strengthen our conclusions about mitochondria. We have redone Figure 4 with this new data. In addition to adding more data points, these new experiments allow us to visualize all 3 mitochondrial markers on the same sample. TOMM20 and VDAC1, known to be localized to the outer mitochondrial membrane, can be seen surrounding MDH2, a mitochondrial matrix protein (Figure 4B). Figure 4 now focuses only on mitochondria and the CNP analysis is presented in Figure 5.

2) For the identification of nodes antibodies could be used.

We tried using a Caspr antibody for this purpose, and while the immunolabel can indeed be seen adjacent to the nodes, in the paranodes, the staining is sparser compared to MBP or PLP and does not define clearly the borders of the nodes.

3) Quantification of mitochondrial markers is done in layer 5, but the other analyses are performed in layer 3 and 4. The analysis could be extended.

We have extended the analysis of mitochondrial markers to include layers 3a, 3b and 4, and we have also extended that analysis of cytoskeletal markers, MBP and PLP to include layer 5, so in this new version the analyses encompass layers 3a through 5.
