# Supplementary material for: Distinctive Structural and Molecular Features of Myelinated Inhibitory Axons in Human Neocortex
Source: eNeuro. 2018 Oct 16;5(5):ENEURO.0297-18.2018. doi: 10.1523/ENEURO.0297-18.2018 (PMC6220577; doi:10.1523/ENEURO.0297-18.2018)
Supplement: Extended Data Table 2-1 — Antibody controls: Pearson’s correlation (PC) coefficients from four different control experiments. The comparison between adjacent sections tests the consistency of staining, as the distribution of targets is very similar on two adjacent ultrathin sections (70-nm thickness). This correlation is influenced by antibody characteristics, but also the size of targets, with smaller targets displaying larger spatial variability from section to section, and therefore a smaller PC coefficient. The comparison with an antibody against an overlapping antigen is a test for the specificity of staining. The following comparisons were done: MBP/PLP, MBP/CNP, MDH2/TOMM20, MDH2/VDAC1. The lower PC coefficient for CNP reflects the fact that CNP is present only in certain regions of the myelin sheath, while MBP is an integral protein of myelin. The antibodies against mitochondrial proteins (MDH2, TOMM20, and VDAC1) also have lower PC coefficients, because TOMM20 and especially VDAC1 antibodies give a rather sparse punctate immunolabeling of mitochondria (Fig. 4; Extended Data Figure 2-2), and even when labeling the same mitochondrion, the immunofluorescence signal from the different antibodies often does not overlap. Another test for specificity is the comparison with an antibody against a spatially exclusive antigen. PC coefficient values of 0 and below are expected in this case. MBP, PLP, and CNP (present in the myelin sheath) were each compared with GABA (inside inhibitory neurons); MDH2, TOMM20, and VDAC1 (mitochondria) were compared with MBP (myelin sheath). And finally, all antibodies were compared with DAPI to control for background nuclear staining. Download Table 2-1, DOCX file. [file sup_enu-eN-NWR-0297-18-s01.docx]

**Table 2-1. Antibody controls:** Pearson’s correlation (PC) coefficients from 4 different control experiments.

| **Control Experiments:** | **Antibody** | | | | | | |
| --- | --- | --- | --- | --- | --- | --- | --- |
|  | | **MPB** | **PLP** | **CNP** | **MDH2** | **TOMM20** | **VDAC1** |
| **Adjacent section** | | 0.91 | 0.96 | 0.36 | 0.45 | 0.31 | 0.20 |
| **Overlapping antigen** | | 0.93 | 0.93 | 0.54 | 0.41 | 0.41 | 0.38 |
| **Exclusive antigen** | | 0.04 | 0.04 | 0.02 | 0.00 | -0.01 | 0.03 |
| **Nuclear** | | -0.08 | -0.1 | -0.04 | 0.00 | -0.02 | 0.01 |
