# Supplementary figures and images for: Distinctive Structural and Molecular Features of Myelinated Inhibitory Axons in Human Neocortex
Source: eNeuro. 2018 Oct 16;5(5):ENEURO.0297-18.2018. doi: 10.1523/ENEURO.0297-18.2018 (PMC6220577; doi:10.1523/ENEURO.0297-18.2018)

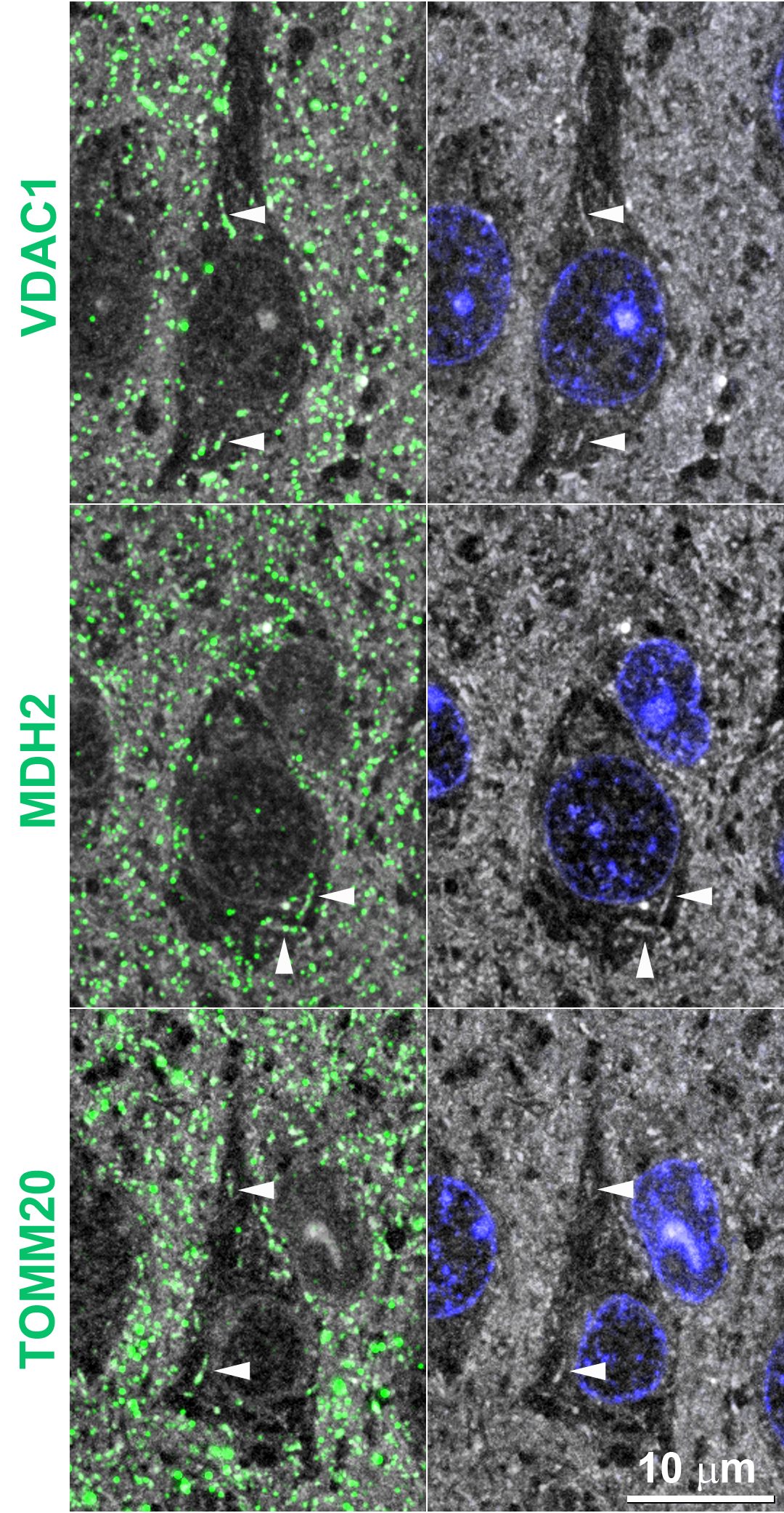

Supplement: Extended Data figure 2-2 — Immunolabeling of mitochondria on LRWhite sections from mouse neocortex. Each image is a MAX projection from four serial sections, 70 nm each. The grey image is the tissue autofluorescence imaged in the 488 channel, and it is superimposed with the immunofluorescence from a mitochondrial marker in green (left column) or with the DAPI label of nuclei in blue (right column). Arrowheads point to several mitochondria, which can be seen as brighter elongated structures within the neuronal cell bodies. There are also abundant mitochondria within the neuropil, but they are harder to distinguish in the autofluorescence image. Download Figure 2-2, TIF file. [file sup_enu-eN-NWR-0297-18-s02.tif]
